# Ethical oversight in impact evaluations: External advisory committees to assess programming risks

**DOI:** 10.1073/pnas.2509773122

**Published:** 2025-11-14

**Authors:** Darin Christensen, Allison N. Grossman, Guy Grossman, Jon Kurtz, Jeremy Weinstein, Jessica Wolff

**Affiliations:** ^a^Department of Public Policy, Luskin School of Public Affairs, University of California, Los Angeles, CA 90095; ^b^Department of Political Science, Tulane University, New Orleans, LA 70118; ^c^Department of Political Science, University of Pennsylvania, Philadelphia, PA 19104; ^d^Research and Learning, Mercy Corps, Washington, DC 20036; ^e^John F. Kennedy School of Government, Harvard University, Cambridge, MA 02138; ^f^Immigration Policy Lab, Stanford University, Stanford, CA 94305

**Keywords:** ethics, impact evaluations, conflict of interests, migration, fragile states

## Abstract

Social scientists not only conduct impact evaluations but also participate in the design and implementation of the programs being evaluated. While Institutional Review Boards (IRBs) oversee research activities, they do not assess risks posed by the interventions themselves. We propose establishing External Advisory Committees (EACs) to provide independent, expert oversight of programming risks. EACs complement IRBs by focusing on potential harms to participants and communities, offering dynamic risk assessments, and advising on program adaptations or termination. By providing impartial expertise, EACs help address potential conflicts of interest that may arise when researchers and implementers are invested in a program’s continuation. We illustrate the value of EACs through our experience implementing a cross-border labor migration program in Niger. Our EAC provided crucial guidance on scaling up the intervention after a pilot study and adapting the program following an unexpected military coup. While EACs introduce additional costs and may limit researcher autonomy, they generate accountability and are particularly valuable for novel and politically sensitive interventions in fragile environments.

The past two decades have seen a dramatic increase in the demand for rigorous impact evaluations to test the effect of programs, projects, and policies.^*^ Impact evaluations can assess effects of novel programs and interventions at scale, thereby generating critical insights for theory and policy (7). At the same time, experimental manipulations of the social, economic, and political world naturally raise thorny ethical issues (8–10), including potential harm to study participants (11), and their communities more broadly (12).^†^

We focus on ethical concerns that arise from researchers’ involvement in the design and implementation of the programs being evaluated. Instead of simply evaluating an existing intervention, researchers are often intimately involved in program design, fundraising, and implementation. Once launched, researchers influence whether these programs should continue or be amended given the risks posed to participants and their families and communities. Given these roles, researchers share responsibility for the potential harm caused by the interventions they helped to launch and oversee.

Yet, researchers lack institutional structures to facilitate impartial deliberations on program termination or adaptation when their own professional incentives encourage them to continue. First, we describe these concerns and explain why they are not easily addressed by existing institutional guardrails tasked with protecting program beneficiaries, such as Institutional Review Boards (IRBs), grant-making Ethics and Society Review boards (ESR), and preregistered stopping rules. Second, we introduce External Advisory Committees (EACs) and explain how and why they can address some of the ethical concerns that arguably cannot be addressed by current institutional guardrails. Third, we demonstrate the utility of EACs using our experience launching an RCT in Niger in collaboration with Mercy Corps, an international NGO. We conclude with a discussion of the conditions in which EACs might be most warranted.

## 1. Problem Statement

In impact evaluations, researchers often influence which subjects receive an intervention. In RCTs, for example, researchers use a coin flip or, more often, a random number generator to assign subjects to the treatment and control arms of the study. While this randomized treatment allocation has attractive statistical properties, it raises an ethical question about whether one should use chance to distribute an intervention with uncertain benefits and harms (13). Past work debates when, if ever, it is justifiable to randomize versus more deterministically allocate (e.g., using a ranking of need or merit) access to a particular intervention (8, 14, 15).

These debates presume that a program exists to be allocated—an organization stands ready to roll out a program and, given its limited resources, enlists researchers to determine who will (initially) gain access. However, researchers now often play a role in designing and fundraising for new programs and monitoring their implementation. We are not just evaluating what would have otherwise happened; we are helping to launch and steer interventions or scale existing programs (16). We welcome this development: Social scientists should draw on past studies and theory to help design promising interventions and rigorously evaluate those innovations. Yet, when researchers participate in the design of interventions, we share in responsibility for the potential resulting harm. Thus, researchers should assess and actively manage risks resulting from participation in the programs we initiate rather than delegating these judgments to implementing partners, which seems to be the current default.

Conflicts of interest, however, compromise researchers’ capacity to independently manage these risks associated with programming. Having invested time (frequently best measured in years) in program design, fundraising, and the associated impact evaluation, we may be convinced of an intervention’s merits and reluctant to overhaul or terminate a program. Moreover, researchers’ career incentives often push toward continuation. In insisting on a program’s termination, we give up the publications we hoped would follow and may sour our relationships with implementing partners or donors who disagree with our risk assessment. In cases where members of the research team are being paid to conduct the evaluation or help to lead or advise the implementing partner, recommending termination could result in immediate loss of income and funding streams. At the same time, conflicts of interest also compromise the ability of implementing organizations to self-regulate programming risks. From their perspective, canceling or making major changes to a program may involve returning unspent funds and laying off staff, including those overseeing the program and monitoring participant harm. Researchers and their implementing partners need a third party to independently assess whether a program poses an undue risk to participants. Such structures are particularly needed for novel and politically sensitive interventions in fragile contexts.

While institutional review boards (IRBs) provide an important form of oversight, they do not serve this role (17). IRBs provide a dynamic assessment of risks posed to human subjects attributable to participation in research activities (18). In the social sciences, IRBs review protocols for recruiting subjects and interviewing or observing those individuals, as well as plans to secure subjects’ privacy. IRB members are researchers with experience conducting trials that can evaluate common risks associated with different types of data collection. Their mandate and expertise do not necessarily enable them to determine whether the program being evaluated poses an undue risk to participants or their neighbors, many of which may not be research subjects (11). Such determinations—whether it is ethical to proceed with a particular program in a particular place and time—should be based on a familiarity with the proposed intervention and a knowledge of the evolving operational environment. However, it is not feasible for a university to set up an IRB with the subject-matter and contextual expertise needed to evaluate the myriad programs and places where affiliated social scientists conduct impact evaluations (19). Moreover, IRBs oversee many research studies in parallel and do not have the capacity to engage in the deep and sustained conversations needed to assess and navigate emerging risks.

If the IRB cannot provide this oversight, we could attempt to tie our own hands. Before any programming, researchers and their implementing partners could publicly commit to a set of stopping rules that trigger the cessation of certain activities (20). Of course, this does not eliminate potential conflicts of interest—rules might be more or less permissive—but public precommitment enables scrutiny and could impose discipline by raising the specter of being perceived to transgress one’s red lines. This is part of the justification for preanalysis plans, which try to address conflicts of interest thought to undermine the replicability of social scientific results. We agree that researchers and their partners should try to enumerate programming risks and ethical guidelines before a program launches. The EAC provides an institutional complement to such efforts, helping to overcome four common challenges that arise in assessing and responding to anticipated and emergent programming risks.

First, ex ante risk assessments focus on foreseeable risks; we do not write rules about risks we did not anticipate. Second, stopping rules force decisions about whether to suspend or discontinue the program as originally designed; they typically do not specify how the program might be adapted. Third, rules are rarely as objective as they seem, and enforcing rules often requires judgment calls: We can debate whether a particular adverse event should be counted or, if we are assessing rates relative to a control group, what critical value we should use to determine if a rule was violated. Given potential conflicts of interest, these judgment calls may lack credibility even when made in good faith.

Finally, in many contexts, we do not have the data needed to implement stopping rules, which require that a program be halted if the intervention generates a statistically discernible increase in certain adverse events. We typically do not want to establish a zero-tolerance stopping rule: Death and hardship happen absent any intervention, and we do not wish to terminate programs because participants face such inevitabilities. Yet, to determine whether the program increases the rate of adverse events we need an estimate of the counterfactual rate (e.g., how many deaths would have happened without the intervention). Many studies run a single, endline survey after programming has already concluded. Even where researchers and implementing organizations collect higher frequency data on program participants, they often do not compile similar data from the control group, which may (by design) have no interactions with the implementing organization. In data-rich contexts (e.g., university health systems), one might be able to estimate this rate using administrative data. In other settings, we must rely on more subjective assessments of whether adverse events are attributable to an intervention. If we cannot trust the assessments of researchers with potentially conflicted interests and this is beyond the scope and expertise of an IRB, how should we proceed?

## 2. EAC Design

We propose that researchers and their implementing partners consider constituting an EAC to dynamically assess and advise on whether an intervention poses an undue risk to participants or their communities. Below we discuss when an EAC is most desirable. While EACs can have different mandates depending on context, they should all adhere to the following principles:

### 2.1. Independence.

To avoid conflicts of interest, members of the EAC should not have a professional stake in the impact evaluation or implementing organization. EAC members may already be acquainted with researchers; people with overlapping subject matter and regional expertise likely inhabit the same epistemic community. However, EAC members should not have strong personal ties that could be perceived to compromise their independence. If EAC members are paid for participation, their compensation should be independent of whether the program is terminated. EAC members should be free to publicly discuss their recommendations while maintaining the privacy of research participants and other EAC members.

### 2.2. Expertise.

EAC’ members should collectively possess regional and subject-matter expertise. The EAC is tasked with assessing whether a specific program in a particular context poses an undue risk to participants or their communities. While this determination can be influenced by information shared by researchers and the implementing organization, it should also draw on EAC members’ outside knowledge of the intervention and/or operational environment. In data-poor contexts, knowledge of conditions on the ground (i.e., having a well-informed prior belief) is necessary to judge whether adverse events should be attributed to the intervention or changes to the operational environment meaningfully affect a program’s risk profile.

These first two principles describe the qualities that teams should consider when recruiting EAC members.

### 2.3. Authority.

The EAC complements oversight by an IRB. While the IRB focuses on risks that arise from participation in research activities, the EAC has a distinct focus on risks that arise due to the intervention, including risks to individuals who are not research subjects or direct beneficiaries of the program being evaluated (e.g., participants’ dependents or neighbors). We also recommend that the EAC considers risks that program and research staff face in delivering, monitoring, and evaluating the program. Enumerators’ occupational safety is not typically within the IRB’s purview, unless these staff are also research subjects (21). While IRBs could theoretically be tasked with mandating EACs for certain projects, this determination would require IRBs to identify when an intervention and context interact to pose risks that call for additional oversight. This is beyond the scope of an IRB and, thus, would require new capacities and expertise.

The EAC should be able to request information from researchers and the implementing organization, including deidentified data or summaries thereof, if permissible under the study’s IRB protocol. The EAC should privately deliberate and provide a written summary of its assessment and any recommendations to the research and implementing organization. While an EAC could be vested with the power to terminate a program, most will serve an advisory role and should be empowered to suggest program adaptations short of termination.

### 2.4. Visibility.

The existence of an EAC and its membership should be noted in any preregistration and in eventual publications. Moreover, in published work, researchers should list and explain any decisions they took that deviated from the EAC’s guidance. This is comparable to the current practice of enumerating deviations from preregistered measurement or estimation procedures. While researchers will, in most cases, retain ultimate decision-making power, they must anticipate that decisions contravening their EAC will be subject to additional scrutiny.

Teams can, of course, opt for even greater transparency (e.g., publishing all recommendations or minutes from EAC meetings) and specify these choices in their EAC charters. We stop short of requiring more extensive disclosure, recognizing that it would likely raise the cost of administering an EAC and may affect the recruitment and deliberations of EAC members.

### 2.5. Dynamic.

As with an IRB, the EAC should convene before the intervention is launched and provide an ongoing review of programming risks. Static risk assessments are insufficient for two reasons. First, impact evaluations tend to study novel interventions. Where we cannot draw on past experiences, it may be challenging to identify all unintended, adverse consequences until the intervention has started to roll out. Second, an intervention deemed safe in one moment may later pose an undue risk if conditions on the ground change. The EAC should set a schedule and end date for periodic reviews. It should also identify events requiring immediate notification of EAC members or emergency meetings.

Those familiar with medical trials will note that an EAC is the social science analog to Data and Safety Monitoring Boards (DSMBs). DSMBs are independent, expert bodies that periodically review data from clinical trials and recommend modifications to the study protocol (including termination) to safeguard participants’ welfare (22). We note two differences. First, clinical trials typically take place in controlled environments. In most instances, DSMBs do not need to consider the political or social context surrounding a particular study site and whether those contextual features affect the risks associated with a specific intervention. Second, DSMBs perform independent analysis of midline data. During a double-blinded study, DSMB members may be the only individuals allowed to unmask participants’ treatment assignments to assess whether (adverse) outcomes differ across treatment arms (23). Many impact evaluations in the social sciences, however, do not collect midline data and cannot passively monitor adverse outcomes in control groups. We, thus, expect EAC’s assessments to be less statistical and more subjective, while being more timely and responsive.

Our proposal also builds on the critique and proposal from (17), who propose conditioning grant funding on review by an ESRs to compel researchers to identify and mitigate risks to society (and not just research subjects). EACs can provide ongoing assessment of societal risks, which may not be entirely foreseeable at the funding stage, and generate accountability even after funding has been awarded. Moreover, while ESRs must consider a wide range of applications, an EAC can enlist members with regional and programmatic expertise related to a specific project. We also see EACs as a complement to the preregistered ethical guidelines proposed by (20). An EAC could help to operationalize these guidelines, providing independent, expert assessment of whether a stopping rule has been triggered, as well as guidance about how program activities might be adapted in response. EACs can also guide how teams respond to unforeseen risks that emerge after preregistration.

## 3. EAC Application: Facilitating Cross-Border Migration from Niger

We established an EAC for a program that we codesigned with Mercy Corps (MC), which has operated in Niger since 2005. The “Planning for Productive Migration” program (PPM) enables legal labor migration by young men to other countries in the Economic Community of West African States (ECOWAS). We created PPM to (1) overcome common barriers to cross-border migration and (2) increase the likelihood that migration contributes to the economic and psycho-social well-being of migrants and their families. The PPM program was first piloted with 110 participants in four communities in February 2022. In consultation with the EAC, which was constituted at the piloting stage, the program was scaled up in June 2023 to 940 participants across 83 communities. We describe the intervention’s context in *SI Appendix*, section 1.

### 3.1. Intervention.

PPM targets young men in Tahoua region between the ages of 18 and 35—the demographic most likely to participate in labor migration (24). The PPM program facilitates safe, legal, and productive migration by relieving constraints that prevent young men from migrating to find higher-paying work. The intervention includes three components.^‡^ First, participants attend eight interactive trainings (over 30 h) to discuss whether migration is the right choice for them and their families and, if so, what preparations they can make to ensure their moves are legal, safe, and productive. Second, trainers visited each participant’s household two times to convene household dialogues. Finally, all participants who completed the training and household dialogues and secured the necessary travel documents and vaccinations were eligible for travel support (roughly $200) that covered a round-trip bus travel to popular destinations within ECOWAS. ECOWAS allows citizens to enter, reside, and work in any member state.^§^

Cross-border migration, while potentially beneficial, carries significant risks across three dimensions. First, migrants face various physical risks, including limited healthcare access, personal safety concerns during travel and settlement, and vulnerability to trafficking networks and exploitation, particularly in fragile states. Second, migrants encounter economic challenges, such as unemployment and labor exploitation, which can prevent them from supporting themselves or sending remittances to families left behind. Our program participants lack the resources to self-insure against such risks and could not anticipate support from a social safety net. Third, migration imposes substantial social and psychological costs through xenophobia, discrimination, and the emotional toll of family separation and disrupted social networks. These various risks affect not only the migrants but can cascade to their households and broader communities of origin, potentially undermining the economic and social benefits that migration might otherwise deliver.

In sum, by facilitating cross-border migration—a new approach to livelihoods support for the implementing organization—PPM introduced a set of new risks to program participants, and by extension, a set of reputational risks for Mercy Corps and its donors. In addition to operating a hotline and emergency fund, establishing the EAC was a central component of the risk-mitigation strategy that we developed to manage these risks and, thus, secure support from key stakeholders and donors. These additional measures are described in *SI Appendix*, section 1B. MC’s staff in Niger implemented all elements of the program. The research team contributed to the program’s design, helped raise funds for implementation, monitored the program’s rollout, and oversaw the randomization and data collection for the impact evaluation.

### 3.2. EAC Composition and Charter.

When constituting our EAC, we sought members with no conflicts of interest, whose collective expertise included the demography and politics of Niger and neighboring countries; the risks, benefits, and barriers to labor migration; and familiarity with impact evaluations. Our EAC comprised five members listed in *SI Appendix*, section 3B. We invited Professor Arsène Brice Bado, an expert in ethics, forced migration, and political instability to chair the committee. All EAC members were paid an upfront honorarium, and their duties were codified in a jointly developed charter (*SI Appendix*, section 3C).

Our project’s EAC was scheduled to meet every three months for the year after the intervention launched, which is when MC planned to end all program activities. Before every EAC meeting, the research team shared a report focusing on two topics. First, the report described changes to the operational environment that could elevate risks for participants, including any campaign of anti-immigrant violence in a destination country or disease outbreaks, political instability, or political violence in Niger or destination countries. Second, the report documented severe adverse events, including the death of a study participant, their spouse, or one of their children or instances of grievous bodily harm or human rights abuses for participants in treatment and control groups. See a report example in *SI Appendix*, section 3E. If the research team or MC learned that a program participant had died, we committed to rapidly reporting this to the EAC and convening, at their request, an emergency meeting (*SI Appendix*, section 3D). As we discuss below, we convened several unscheduled meetings of the EAC in response to unanticipated political upheaval in Niger.

EAC meetings started with an open session attended by one or more members of the research team and MC. This allowed the EAC to pose questions about the report they received or other aspects of the intervention or operational environment. The EAC members then deliberated in a second closed session and shared recommendations in writing. We did not specify how the EAC should resolve conflicting viewpoints among members (e.g., a voting rule) and did not ask them to attribute particular recommendations or viewpoints to specific members. We provide an example of the EAC’s recommendations in *SI Appendix*, section 3F.

### 3.3. EAC’s Role in Consequential Programming Decisions.

Beyond the ongoing monitoring of adverse events described above, we emphasize two moments—one foreseen, the other unexpected—in which we faced consequential decisions about whether and how to continue the PPM program. In these moments, the EAC provided invaluable advice about how to proceed.

#### 3.3.1. Scaling up.

In 2022, we piloted the PPM program with 210 men from four communities in Tahoua, randomly assigning 110 to the program and the rest to a control group. The pilot was not designed to test efficacy but rather program delivery, and risk-mitigation protocols, including our ability to maintain contact with a mobile population. During the pilot, one PPM participant died while in Abidjan, prompting a visit by MC staff to the family to express condolences and explore the cause of death. We sent notice of this adverse event to the EAC (*SI Appendix*, section 3D), which convened an unscheduled meeting promptly. The research team presented data showing no health differences between treatment groups. The EAC found the death unrelated to the program and advised reinforcing hotline access and establishing a tighter health emergency protocol.

We conducted an endline survey in October 2022 with all pilot subjects. The EAC convened in early December to discuss our report that compared economic, migration, and health outcomes for individuals randomly assigned to the PPM program vs. the control group. Our report also included a summary of changes to the risk environment for participants (there were none) and severe adverse events. After deliberating, the EAC recommended that we scale the program to conduct a full-scale RCT as the pilot indicated potential significant benefits and no major risks (see *SI Appendix*, section 2 for additional details).

#### 3.3.2. Adapting to unexpected political instability.

We recruited 3,000 households for the RCT and completed a baseline survey in June 2023. Programming launched in July and was to continue through October; risk mitigation measures would stay in place for an entire year.

However, on July 26, high-ranking members of the Nigerien military staged a coup d’état, ousting and holding captive the country’s democratically elected president. This political upheaval was surprising: Niger was viewed as a “bastion of stability in the Sahel” and a reliable partner for Western governments.^¶^ In response, ECOWAS threatened military intervention and imposed sanctions, which involved the official closure of Niger’s borders with Nigeria and Benin. (The borders to Burkina Faso and Mali—ECOWAS members run by military governments sympathetic to the junta—remained officially open.) While there were demonstrations in Niamey, we received no reports of unrest in Tahoua, which is a 10+-hour drive from the capital. The coup changed the operational environment for the program primarily by potentially limiting opportunities for regular cross-border migration to the most popular destinations in ECOWAS and by interrupting trade, thus increasing local food prices.

Following the coup, we worked with the EAC to address two questions: First, under what conditions should we terminate the program; second, if we proceed, how should we change the program? We proposed a set of four criteria for stopping the program: 1) large-scale violence in Niger due to a foreign intervention or civil war; 2) significant worsening of the security situation in the region due to terrorism or counterterrorism; 3) MC shuts down operations or cannot safely deliver the program; and 4) all borders to ECOWAS countries are closed, both de jure and de facto. If none of these criteria applied, we proposed a contingent plan for adapting programming in response to different scenarios—whether borders could be crossed without the risk of harassment or detention and whether Nigeriens faced hostility in destination countries. The EAC endorsed these decision-making protocols and recommended meeting every two weeks to review whether the program should be stopped or changed in response to the new risk environment.

To inform these meetings, we gathered additional information on the status of borders: Enumerators visited popular border crossings (with Benin, Burkina Faso, Mali, and Nigeria) every two weeks to observe whether Nigeriens could cross safely and without harassment, and we monitored a reputable bus line to determine which routes they continued to operate. We also regularly reviewed security briefs and monitored regional news outlets for stories about organized violence or harassment directed at Nigeriens. These sources were augmented by the expertise of our EAC members, most of whom live in ECOWAS.

After deliberating, the EAC endorsed the resumption of training and household dialogues in Tahoua. However, we delayed providing travel support, initially planned as a paid bus ticket to the participant’s chosen destination within ECOWAS, while we gathered more information on border crossings and the regional security situation.^#^ By November, Niger remained under military rule, but the risk of conflict appeared negligible; social unrest was confined mainly to Niamey and, even there, was relatively muted. In monitoring border crossings, we observed that Nigeriens were moving in large numbers into Benin and Nigeria. While these borders were officially closed, individuals freely crossed in full view of border agents by either walking across the border to Nigeria or taking a short canoe (*pirogue*) ride over the Niger River to Benin. Having completed the required training and household dialogues, program participants were eager to receive the travel support they had been promised; labor migrants typically embark late in the year after the harvest.

To honor promises made to participants, including preserving their agency and well-being, we proposed a programming change to the EAC: Rather than issuing bus tickets, MC would instead provide a cash transfer of roughly equivalent value. Our participants overwhelmingly planned to migrate to Côte d’Ivoire, and the most direct route involved crossing into Benin. A reputable bus carrier continued to sell tickets from Tahoua to Côte d’Ivoire. Still, its passengers took one bus to the Niger side of the border, disembarked and separately purchased crossing on an unaffiliated canoe, and then boarded a second bus from the same carrier waiting in Benin.^‖^ Neither MC nor the EAC regarded the canoe crossing as an undue risk to participants. However, MC did not feel it could provide a ticket requiring passengers to use an unregulated mode of transport for part of the journey. A cash transfer instead allows participants to make their risk–benefit calculations and use the cash accordingly—to support planned migration or to stay in Niger. This cash transfer would be disbursed only after an extra training session that provided up-to-date information on major border crossings and reiterated the risks of migration to Mali and countries outside of ECOWAS.^**^

We convened two EAC meetings to discuss this proposal and feasible alternatives. After deliberating, the EAC endorsed our proposal. The EAC recommended that we continue monitoring border crossings and notify participants of any changes.

The coup, an unforeseen event, underscores the value of the dynamic, expert, and independent review provided by an EAC. The coup did not meaningfully change the risks associated with surveying, so it did not affect our IRBs’ assessments. It was an event that was unlikely to have been considered in an ex ante risk assessment or encoded in a stopping rule. However, in consultation with our EAC, we ultimately decided against stopping the program. Amid rising prices and food insecurity, we felt that continuing to provide financial support, albeit in an alternative form, best preserved participants’ agency and well-being. The EAC’s impartiality allayed concerns—including among donors who were contemplating pulling funds from our program and others in Niger—that this decision to continue and adapt the program was driven by the potentially conflicting interests of the researchers and implementers.

## 4 Discussion

When researchers participate in the design and implementation of interventions, they assume some responsibility for the risks posed to participants, their families, and society. Our professional incentives and commitments to partners and donors can color our assessments of these risks, and we should seek out impartial experts to help surface and resolve conflicting views. The scope of the IRB is too narrow to serve this function, and it can be difficult or unwise to tie our hands with strict and static stopping rules. After several years of scoping research and a pilot study in Niger, we still failed to anticipate major political events that shifted the risk environment of the PPM program.

In forming an EAC, researchers commit to ongoing tracking and reporting on risks. Even if the EAC cannot force decisions, it requires transparency. We must weigh intervention’s evolving benefits and harms and justify our choices to continue or adapt programming to an independent body of experts. This ethical oversight generates accountability. Below we provide some final reflections for those considering setting up EACs to accompany their impact evaluation.

### 4.1. What Are the Costs of Creating an EAC?

While honorariums paid to EAC members are the most obvious cost, they are not necessarily the largest. Researchers and the implementing organization may need to collect additional data to provide informative reports to the EAC. Moreover, the EAC may create unanticipated demands for information: In our Niger project, for example, we had not planned to hire enumerators to visit multiple border crossings regularly. Information on adverse events and the risk environment must be periodically summarized in reports to the EAC, and researchers and their implementing partners must be available to brief the EAC and answer members’ questions. The research and implementation teams would have needed to undertake much of this additional assessment to inform program decisions even without an EAC in place. However, the heightened requirement to collect and report on the context changes and risks to the EAC provided a greater level of accountability for doing so. We estimate spending just under one percent of our project’s budget on the EAC, including honoraria, data collection and analysis, and administrative support. We hope interested researchers can secure grant funding to offset these costs and that, over time, funding agencies will allow or even encourage adding these costs to funding requests. However, we recognize that research resources are inequitably distributed. We do not advocate making EACs a requirement, at least until funding norms change and evolve, partially because this would disadvantage scholars with fewer resources. We note, however, that scholars can economize by not paying honoraria to EAC members; researchers are accustomed to providing unfunded mentorship and service to peers, for example, by reviewing grant applications or advising on tenure cases.

An EAC also limits implementers’ and researchers’ autonomy. Suppose the EAC disagrees with researchers and implementers and provides a conflicting recommendation. They could heed the EAC and, at a minimum, incur a psychic cost for taking an action they disagree with. Alternatively, the researchers and implementers could defy the EAC’s recommendation, which is not binding. Yet, they assume reputational risks by rejecting the advice of an expert body they publicly constituted to provide ethical oversight. Per the “Visibility” principle, the team should document any decisions that deviate from their EAC’s recommendations in published work to facilitate scrutiny. This is by design: If there were no cost to ignoring your EAC, then it would be window dressing and not a real source of accountability.

### 4.2. Which Projects Benefit from an EAC?

We appreciate that our proposal may sound demanding. Researchers already report to IRBs, donors, and their peers. Should we be subjected to more reviews? Can we not be trusted to police the programs we evaluate? We believe that only some impact evaluations need an EAC. An EAC will be especially valuable in three scenarios: First, for novel interventions where the potential harms are nontrivial and challenging to foresee, in fragile operational environments, and when supporting interventions that are potentially politically sensitive. In such instances, conducting ongoing risk monitoring and assessing whether a program needs to be adapted in response to unanticipated harms or changes in the operational environment is more important. Finally, an EAC addresses conflicts of interest that could compromise the researchers’ ability to properly balance participant risk against the benefits of adhering to the original implementation plan. If these conflicts are not present or are addressed by other mechanisms, then it may not be necessary to constitute an EAC to scrutinize programming decisions.

Simply establishing an EAC will not bring these benefits. Our experience illustrates key elements that researchers and implementers need to put in place for EACs to play an effective advisory function. First is clarity on what roles are expected of the EAC. We provided this information upfront via a clear charter, along with an orientation for each EAC member on the committee’s purpose. Then, in advance of each EAC meeting, we clarified the specific risks and decisions we needed their expert advice on. Second is information to inform the EAC’s advising and our subsequent decisions. Putting a standard operating procedure in place for monitoring and responding to severe adverse events and other risks allowed us to inform and get timely responses from the EAC. We found two main data sources to be critical for this: routine surveys to monitor potential harms, including among the control group, and a dedicated hotline to capture idiosyncratic events among program and research participants. Third is the ability to adapt programming based on the EAC’s counsel. Processes within Mercy Corps, and flexibility by the donors to the PPM program, enabled us to quickly pivot major program activities—namely shift to provide cash transfers following the coup in Niger. Such flexibility is not a given in many international development programs. Yet such flexibility is essential for acting on EAC recommendations that require significant program changes.

EACs provide ethical oversight for impact evaluations designed and implemented by researchers to test novel interventions in fragile contexts. While they introduce additional costs and complexity, EACs provide three critical functions that existing institutional structures do not adequately address. First, they offer dynamic, context-sensitive oversight of programming risks that complements the more narrowly focused review of research activities by IRBs. Second, they help resolve conflicts of interest by providing independent expert guidance when researchers and implementing partners face difficult decisions about continuing, adapting, or terminating interventions. Third, they create accountability through regular monitoring and reporting requirements, even after funding has been secured and programming has begun. Though our focus is on program evaluation, EACs could prove valuable in other research settings where standard institutional oversight may lack the specialized knowledge needed to assess context-specific risks to participants.

We emphasize here that an EAC can lighten the moral burden that researchers and implementers feel when they initiate an intervention with uncertain benefits and harms. Rather than unilaterally contemplating decisions that could harm others, researchers and the implementing organization benefit from the counsel of independent experts. They can move forward with greater confidence and accountability knowing that an EAC agreed with their choices. As social scientists increasingly participate in program design and implementation, establishing EACs helps ensure we meet our ethical obligations to participants and their communities while maintaining the scientific integrity of our research. We believe the framework we propose here—including clear principles for independence, authority, expertise, and dynamic review—can serve as a model.

## 5. Materials and Methods

Stanford University IRB (protocol #67651), Tulane University IRB (protocol #2024-296), UCLA IRB (protocol #22-001731), and the University of Pennsylvania IRB (protocol #852431) approved the full study protocol, including the impact evaluation of Mercy Corps’s programs in Niger. We obtained informed consent from every respondent before every survey round. Consent was provided orally in respondents’ preferred language (French or the local language, Haoussa) per our IRB protocol.

## Supplementary Material

Appendix 01 (PDF)

## Data Availability

There are no data underlying this work.

## Footnotes

^*^Randomized Controlled Trials (RCTs) in field settings, for example, are commonly used across social scientific fields of inquiry, including political science (1), economics (2), sociology (3), operational management (4) and behavioral sciences more generally (5, 6).

^†^The literature concerned with impact evaluations’ ethics focuses almost exclusively on RCTs. However, similar concerns arise when programs are designed with eligibility thresholds, and an regression discontinuity designis used to evaluate its impact around those thresholds.

^‡^Our analysis plan for the associated impact evaluation provides more details on the components of the intervention: https://osf.io/yz56e.

^§^Niger officially exited ECOWAS in January 2025 but was a member of ECOWAS during program implementation and in the year following implementation.

^¶^https://www.dw.com/en/niger-coup/a-66372043.

^#^Recall, participants were only eligible for financial support if they completed at least six trainings and one household dialogue and secured required travel and vaccination documents. Moreover, we refused to support travel to Mali due to ongoing civil conflict.

^‖^Our enumerators observed over a hundred boats ferrying individuals across the border at this crossing. The boat ride took roughly five minutes bank-to-bank.

^**^We had recently surveyed the individuals eligible for a cash transfer, and almost none expressed interest in migrating to high-risk destinations including Libya and other countries outside of ECOWAS. In our pilot, less than three percent of PPM participants migrated to such countries.
