# Supplementary material for: Ethical oversight in impact evaluations: External advisory committees to assess programming risks
Source: Proc Natl Acad Sci U S A. 2025 Nov 14;122(47):e2509773122. doi: 10.1073/pnas.2509773122 (PMC12663984; doi:10.1073/pnas.2509773122)
Supplement: Supplementary file 1 — Appendix 01 (PDF) [file pnas.2509773122.sapp.pdf]

Supporting Information for

Ethical Oversight in Impact Evaluations: External Advisory Committees to Assess Programming Risks

Darin Christensen, Allison N. Grossman, Guy Grossman, Jon Kurtz, Jeremy Weinstein, Jessica Wolf

Darin Christensen.
E-mail: darinc@luskin.ucla.edu

This PDF file includes:

Supporting text
Fig. S1
SI References

Supporting Information Text

1. Context and Intervention

A. Migration in Niger. Niger is one of the poorest countries in the world. Less than half of the population between the ages of 15 and 64 have work (1). Young people have few economic opportunities, particularly in rural areas. Environmental factors, including climate change and desertification, are increasing the competition for land and undermining the long-term viability of weak rural economies. While agriculture employs 78% of Nigeriens, most young Nigeriens aspire to work in other sectors (2).

Cross-border migration could help to satisfy these aspirations and enable rural households to diversify their livelihoods. As part of our scoping research, we surveyed 1,198 households in Tahoua Province (our study area) in April 2021. We found that 67% of young men (18-35) would like to migrate in the next three years, and 74% of those prefer to move outside of Niger.* Yet many will remain stuck: across our sample, we record 664 respondents who deferred or canceled their migration plans, with insufficient funds (67%) and household responsibilities (24%) being the most common reasons. These data suggest that cross-border migration rates would be higher without financial and family constraints. Second, migration would be more productive with additional planning. Among the young men interested in cross-border migration, a large majority were somewhat or very worried about finding a job, finding housing, and avoiding discrimination and harassment. We hypothesize these men would have more success integrating if they received support identifying job opportunities, mitigating migration risks, consulting with family, and paying for travel. Moreover, better communication within households could overcome opposition from family members and improve outcomes for those who stay behind.

Cross-border migration, while potentially beneficial, carries significant risks across three dimensions. First, migrants face various physical risks, including limited healthcare access, personal safety concerns during travel and settlement, and vulnerability to trafficking networks and exploitation, particularly in fragile states. Second, migrants encounter economic challenges, such as unemployment and labor exploitation, which can prevent them from supporting themselves or sending remittances to families left behind. Third, migration imposes substantial social and psychological costs through xenophobia, discrimination, and the emotional toll of family separation and disrupted social networks. These various risks affect not only the migrants themselves but can cascade to their households and broader communities of origin, potentially undermining the economic and social benefits that migration might otherwise deliver.

B. Risk mitigation measures. In addition to information imparted during the training about safe migration and supportive services in destinations (e.g., consulates and diaspora groups), we employed two other strategies to monitor and mitigate risks. We set up a hotline that participants could call 24/7 to receive information or assistance. We also set aside an emergency fund to support participants who could not return home independently. Given low literacy rates, the interactive hotline with recordings in French and Hausa served as a reference for participants. Program staff monitored all calls to the hotline and followed up on requests for assistance. Emergency funds were ultimately not used during the pilot or RCT as there were no severe adverse events that required this support.

2. EAC role in scale up

During the pilot, one participant in the PPM program died while in Abidjan, Côte d'Ivoire. MC learned of this death on September 18, and program staff visited the participant's family on September 20 to express condolences. During this visit, MC learned that the participant had fallen ill with malaria and had been hospitalized for three days before passing. We sent notice of this severe adverse event to the EAC on September 26 (see SI Section D), which convened an unscheduled meeting two days later. In the open session of the EAC meeting, the research team shared more detailed information about the deceased participant's involvement in the PPM program and a comparison of health outcomes for PPM participants vs. control, which found no systematic differences. After deliberating in a closed session, the EAC judged that the severe adverse event was not attributable to the PPM program: the individual had not completed the training or received financial support from MC to migrate; moreover, the individual received medical care, suggesting that migrating did not prevent them from accessing healthcare. The EAC also recommended reminding participants about the hotline, which an acquaintance of the deceased used to inform MC about the death, and establishing a protocol for when and how to support participants facing health emergencies that does not create moral hazard.

We conducted an endline survey in October 2022 with all pilot subjects. The EAC convened in early December to compare economic, migration, and health outcomes for individuals randomly assigned to the PPM program vs. the control group. Cross-border migration was 8 percentage points higher in the PPM group, which also had higher levels of average income and food security. There were no meaningful differences in mental or physical health. Given the small scale of the pilot, none of these differences could be precisely estimated. Our report also included a summary of changes to the risk environment for participants (there were none) and severe adverse events (the one death mentioned above). After deliberating, the EAC recommended that we scale the program to conduct a full-scale RCT as the pilot indicated potential significant benefits and no major risks.

While the pilot's endline data was instructive, we stress that this was a largely subjective determination: our pilot was done in a convenience sample of four localities across two communes. The proposed footprint of the full-scale RCT was 142 new localities spread across eight communes. The programming and evaluation would directly involve 15 times more households. Moreover, some border closures related to the COVID-19 pandemic were still in place during our pilot, which likely constrained

*Of those who said they were not interested in migration, over half said they would like to move but were "uninterested" because they did not think moving was possible.

out-migration. The EAC provided an independent and expert perspective on whether we had developed the protocols and capacity needed to monitor and manage risks for many more migrants — a question outside the scope of an IRB.

3. External Advisor Committee

A. Comparison to DSMBs. DSMBs are independent, expert bodies that periodically review data from clinical trials and recommend modifications to the study protocol (including termination) to safeguard participants' welfare (3). We note two differences. First, clinical trials typically take place in controlled environments. In most instances, DSMBs do not need to consider the political or social context surrounding a particular study site and whether those contextual features affect the risks associated with a specific intervention. Second, DSMBs perform independent analysis of midline data. During a double-blinded study, DSMB members may be the only individuals allowed to unmask participants' treatment status to assess whether (adverse) outcomes differ across treatment arms (4). While we certainly would not discourage an EAC from conducting comparable analysis, we recognize that many impact evaluations in the social sciences do not collect midline data and cannot passively monitor adverse outcomes in control groups. We, thus, expect EAC's assessments to be less statistical and more subjective.

B. Membership.

- **Prof. Arsène Brice Bado** (Chair), Vice President for Academic Affairs, CERAP/Jesuit University, Abidjan, Ivory Coast.
- **Dr. Joseph Asunka**, CEO, Afrobarometer, Ghana.
- **Abdoulaye Harouna**, Experienced humanitarian professional in Niger.
- **Dr. Karen Levy**, Co-Founder, Fit for Purpose, Kenya (formerly Evidence Action with work on No Lean Season)
- **Dr. Oreva Olakpe**, Researcher, Toronto Metropolitan University (expertise on cross-border migration in ECOWAS).

C. Charter.

Proposed Charter of the External Advisory Committee (EAC)

I. Introduction

The purpose of this document is to describe the roles and responsibilities of the independent External Advisory Committee (EAC), a body which provides guidance to the implementation and research teams implementing the Planning for Productive Migration (PPM) program in Niger.

The PPM program addresses constraints that impede legal migration. These constraints include lack of information, travel documentation, social networks, and financial resources. Through training and household counseling, the program helps participants and their families think through the potential benefits and costs of migration. If a participant chooses to migrate, the program provides for round-trip travel to their desired destination in the ECOWAS region. To be eligible, individuals must be men between aged 18–35 who express an interest in migration prior to enrollment in the PPM program. Whether or not they migrate is a choice each participant makes with their family after the training and household counseling are complete.

The PPM program will be piloted with a sample of 110 households in Tahoua, Niger in February 2022, each with a primary young male participant. Mercy Corps will implement the program with research support from the Immigration Policy Lab at Stanford University.[1] Participating individuals will receive the training/counseling program and then be surveyed regularly for six months to monitor their outcomes. In addition, 100 households will be assigned to a "control" group, which will also be monitored over the same six-month period. We will randomly assign which households receive an invitation to the PPM program and which do not. The pilot will inform a potential scale-up to a fully-powered randomized controlled trial (RCT) in late 2022.

This document includes information about the timing and format of EAC meetings, the methods of communicating information to and from the EAC, and the relationship between the EAC and other parties.

II. Roles and responsibilities

The aim of the EAC is to safeguard the interests of program participants and their households and advise the PIs and Mercy Corps about the ethics and credibility of the research study. In addition, because the program is still in development and the pilot is a learning opportunity to inform a fully-powered RCT, the EAC is invited to share feedback and perspective regarding potential program changes going forward.

The EAC will receive regular reports on the pilot. After reviewing and discussing each report, the EAC will advise the internal advisory group (IAG; see Footnote 1 for a list of members) whether, in their view: (i) the pilot should proceed without adjustment; (ii) adjustments should be made to the program design or implementation plan; or (iii) the implementation of the program/trial should be paused or ended given

[1] Principal investigators: Jeremy Weinstein (Stanford); Co-Principal Investigators: Darin Christensen (UCLA), Allison Grossman (Stanford), Guy Grossman (University of Pennsylvania), Beza Tesfaye (Mercy Corps), and Jessica Wolff (Stanford). We refer to this set of individuals as the PIs. The research and program implementation are overseen by an internal advisory group (IAG) that includes [Robert Lankenau (Mercy Corps), Siaka Millogo (Mercy Corps), Jon Kurtz (Mercy Corps), Beza Tesfaye (Mercy Corps), Jeremy Weinstein (IPL), and Jessica Wolff (IPL)].].

changes in the operational environment and/or the frequency of severe adverse events. The EAC can also request additional information from the program implementation and research team.

Based on reports provided to them (and any outside sources of information), the specific roles of the EAC include:

- Evaluating developments that pose additional risks to program participants;
- Assessing the frequency of severe adverse events and evidence of possible harm to participants or their households from the PPM program;
- Deciding whether to recommend adjustments to the program design or implementation plan on the basis of changes in the operational environment or assessments of harm;
- Deciding whether to recommend that the program be terminated either for all participants or for some subset of participants based on changes in the operational environment or assessments of harm.
- Offering input to inform potential changes to the program for the fully-powered RCT.

Recognizing that this is a new and evolving institutional mechanism, the members of the EAC may want to amend their roles and responsibilities or make changes to the organizational structure and decision-making approach of the body. Any proposed changes to the structure and approach should be transmitted by the Chair of the EAC to the program implementation and research team for discussion and consideration. Changes will be made on mutual agreement of the EAC members and the implementation and research team, and a revised charter will be circulated.

III. Composition

The EAC will include five members. The EAC will be composed of a diversity of voices and expertise including, at a minimum: (i) scholars/social scientists from the region in which the program will be implemented (West Africa); (ii) community advocates, likely drawn from development or humanitarian organizations in the region, who are also familiar with research; (iii) domain experts who have conducted research on migration in the region; and (iv) field experimentalists who have worked on RCTs in the Global South. The individuals participating on the EAC will be independent of the research and implementation team and any conflicts of interest will be declared in advance of the launch of the EAC.

The EAC members will include: Harouna Abdoulaye (COPAVE), Joseph Asunka (Afrobarometer), Arsene Brice Bado (CERAP), Karen Levy (Fit for Purpose), and Oreva Olakpe (Ryerson University).

One EAC member, Professor Bado, will serve as chair. He/she will facilitate the EAC meetings and summarize the discussions. The EAC will operate in English but simultaneous translation will be available for any members who prefer to participate in French.

EAC members are expected to operate in good faith with respect to charter of the EAC and to engage in thoughtful and productive deliberation with their colleagues. If the chair determines that an EAC member is engaging in ways that are disruptive of the group's operations, he/she will raise their concerns with the member. If the behavior continues, the chair has the authority to dismiss a member, which he/she will then report to the IAG.

The senior program manager from the Immigration Policy Lab will staff the EAC, organizing and coordinating the meeting schedule.

Members of the IAG, including the PIs and representatives from Mercy Corps, will be available to attend open sessions of the EAC.

IV. Relationships

The EAC will act in an advisory role to the IAG, which governs research and implementation team. The EAC will not make decisions about the program or the data collection. Instead, the EAC will provide recommendations to inform the decision-making of the research and implementation team.

Members of the EAC will be compensated for their time and effort. This will take the form of an honorarium of $3,500, which will be paid out in monthly installments for the seven month duration of the EAC.

V. Organization of EAC meetings

The EAC will hold its first meeting in March 2022. The first ninety minute meeting will focus on (i) familiarizing the EAC with the design of the program and the risk-mitigation protocols and (ii) seeking input on the developments and severe adverse events that should be tracked throughout the pilot. The EAC will review and provide feedback on a template for the reports that will be provided by the research and implementation team to enable EAC discussions. The first meeting will also provide an opportunity for EAC members to seek clarification on their roles and responsibilities and to agree on how their meetings will be run going forward.

Once the program launches and for the six months that subjects are surveyed, the EAC will meet every month. An exceptional meeting would be called immediately in the event of the death of any participant (including both the treatment and control group). Three days in advance of each meeting, the research and implementation team will share a written report with information on developments that pose additional risks and severe adverse events if any such events have been experienced by the program participants or those in the control group. Representatives of the IAG will join the first ten minutes of each EAC meeting to answer any questions. After this, the EAC will meet in closed session to reflect on any new developments and form their recommendation for the IAG.

VI. Documentation and procedures to ensure confidentiality and proper communication

The research and implementation team will report on new risks and severe adverse events in a standardized format. This format will be briefed to the EAC at its organizational meeting and input will sought to ensure it is maximally informative. These reports will draw on information collected by the research and implementation team and will be made available to PIs, IAG, and the EAC.

The EAC will report its recommendations in writing to the IAG in English. These recommendations will also be shared with all PIs and implementation team members. For documentation purposes, the EAC should issue a written report even if no changes to the program or research protocol are recommended.

VII. Decision making

In its deliberations, the EAC can decide to advance a number of possible recommendations including:

- Signaling that no action is needed and the program implementation and research protocol can continue as planned;
- Proposing changes to the program model or research protocol to address new risks or to mitigate potential harms for the ongoing pilot;
- Recommending a pause in pilot program implementation or the research protocol while time is taken to investigate/address a potential concern;
- Recommending that the pilot program implementation or research protocol be halted (for all or for some participants) based on changes to the operational environment or the frequency of severe adverse events.

In addition, the EAC can make suggestions or proposals as to how the pilot program should be changed or adapted in advance of the fully-powered RCT.

In arriving at their recommendation, the EAC should strive for consensus and take a vote only if necessary. Before arriving at a recommendation, the EAC can seek further input from the research and implementation team, either in written form or via a direct conversation. If a vote is taken, the results of the vote should be recorded alongside the recommendation that is conveyed to the Advisory Group.

Effort should be made for all EAC members to attend. The Senior Program Manager at IPL will identify a time that works consistently for EAC members.

Recognizing that EAC members may sometimes be unable to attend, members may share input/feedback with the Chair in writing in response to a written report provided in advance. If a member misses more than two meetings in a row, the Chair should ask the member whether they wish to remain a member of the EAC. If they cannot commit to regular attendance, they should be replaced.

If the EAC is considering recommending major action after a meeting, the Chair should talk with any absent members to check that they agree. If they do not, the decision should be discussed at a subsequent meeting when all members are present.

The EAC will deliberate on the implications of changes to the risk environment and severe adverse events experienced by program participants. Although these criteria have not been finalized, the examples below identify the kinds of developments that will be reported to the EAC in advance of every meeting. These criteria will be further developed and finalized with the EAC.

Risk Environment/Severe Adverse Events: In advance of the EAC meetings, the implementation and research team will circulate a report detailing any significant changes to the risk environment for participants and whether the following severe adverse events have occurred and the proposed actions in response. Case specific details will be provided in order to help the EAC make a judgement regarding whether the event is attributable to the treatment. Any death to a participant would be a cause for an immediate discussion with the EAC within twenty-four hours.

Changes to the Risk Environment	Potential Actions

A campaign of targeted anti-immigrant violence (destination location)	Update participants on risks. Stop subsidizing travel to the destination. Encourage people to return.
Severe outbreaks of COVID in Niger or destination countries – e.g. hospitalization and death.	Update people on risks.
Severe political instability and political violence/civil conflict.	Update people on risks both with respect to destination and transit routes. In the event of civil war breaking out, stop subsidizing travel and reach out to offer return.

Severe Events	Measurement Strategy	Baseline Rate
Death of the participant, grievous bodily harm	Check-in calls. Hotline.	Comparison to the control group.
Death of the subject's spouse or child	Check-in calls. Hotline.	Comparison to the control group.
Wife/family loses home or land	Check-in calls. Hotline.	Comparison to the control group.
Participant experiences severe human rights abuses in destination (trafficking, torture, etc.)	Check-in calls. Hotline.	Comparison to the control group.

While the research and implementation team will report on the risk environment and the frequency of severe adverse events in comparison to the control group, no formal stopping rule will be established in advance. Given the small sample size of the pilot, the EAC will be empowered to evaluate the specifics of the harms that are experienced in light of the baseline rate and knowledge of the context/region and to then make a recommendation to the research and implementation team.

In addition, the research and implementation team will also report to the EAC any anonymous feedback provided by program participants through the Mercy Corps Community Accountability Reporting Mechanism (CARM) or directly to the Institutional Review Boards (IRBs) of the three participating institutions.

VIII. Reporting

The EAC will report its recommendation in a letter to the Advisory Group. The letter can be sent over email and should be provided within three business days. The PPM Internal Advisory Group will review the recommendation and respond with their decision and planned actions within five business days.

Minutes of the meeting will be kept by the Chair. These minutes are only for the internal use of the EAC and will not be shared with the Advisory Group.

IX. After the trial

The role of the EAC will be described in the main report of the study results. This section will include the names and affiliations of the EAC members, unless they explicitly request otherwise. A brief summary of the timing and conclusions of the EAC meetings will be included in an appendix to the paper.

The research and implementation team will provide EAC members with the opportunity to read and comment on any discussion of the EAC's role in draft publications before submission.

EAC members may not share any confidential information on the program, study details, or their confidential deliberations until the primary results have been published.

D. Reporting Protocol for Severe Adverse Events. The EAC's charter envisions reports on significant changes to the risk environment and severe adverse events before scheduled EAC meetings. The reports include case-specific information and any proposed responses.

In the case of a participant's death, we committed to immediately collecting and sharing case-specific information (e.g., cause of death) with the EAC and facilitating an off-schedule meeting. For example, on September 26, we sent the notice below (see SI Figure S1):

Fig. S1. Example Report of Severe Adverse Event

Notice of a Severe Adverse Event in the PPM Pilot
Date: September 26, 2022

Description of Severe Adverse Event

On Sunday, September 18th, the PPM team was informed of the death of an individual (hereafter, Participant A) assigned to the treatment group. Two calls were made to the PPM WhatsApp hotline to share the news directly with the program staff: one call by a member of the treatment group and one by a member of the control group.

Both calls to the hotline included the following details:

- Participant A passed away on Thursday, September 15th in Abidjan, Côte d'Ivoire.
- Participant A was sick with malaria and was only sick for a short time.

On Tuesday, September 20th, the PPM Program Manager visited Participant A's village and family to share condolences. He learned that Participant A sought care at the hospital in Abidjan and was there for 3 days before he passed away.

The EAC then reviewed:

- A report on the participant and his family, drawing on prior data collection as well as MC's visit to the household.
- Data on health outcomes comparing participants in the PPM group to the control group throughout the pilot.

The EAC judged that the severe adverse event was not a consequence of the PPM treatment, as (a) the individual had not completed the program or received a bus ticket (he had only attended the first week of training) and (b) the individual had sought medical care in Abidjan for malaria but unfortunately succumbed to the disease (so being abroad did not limit access to medical care).

E. Example: Report to EAC.

Planning for Productive Migration Pilot: External Advisory Group
Monthly Report

Meeting Date: July 7, 2022
Reporting Dates: June 3 - June 29, 2022

1. PROGRAM STATUS

**PPM Training status**	Pilot program training in Illela & Keita communes complete
**WhatsApp hotline**	Active & in use
**Total participants**	110 treatment households; 100 control households
**Survey wave**	Round 3 completed in June
**Total # of bus tickets provided overall**	18 outbound tickets tickets overall
**Total # of bus tickets provided in the last period**	2 outbound tickets
**Country destinations currently supported by the program (open land borders)**	(see table below)

**Country destinations**	**Open land borders (Yes/No)**
Côte d'Ivoire	No
Togo	Yes
Nigeria	Yes
Benin	Yes
Ghana	Yes
Mali	No
Burkina Faso	Yes
Sénégal	Yes
Cap Vert	Yes
Gambie	Yes
Guinée Bissau	Yes
Sierra Leone	Yes
Guinée	Yes
Libéria	Yes

**Monthly survey respondents**	102 Primary participants:<br>- 54/98 in control<br>- 48/110 in treatment
**Countries with treatment & control participants in reporting period according to monthly phone surveys**	(see table below)

Control group	Treatment group
Niger (51)<br>Côte d'Ivoire (9)<br>Central African Republic (1)<br>Libya (3)	Niger (45)<br>Côte d'Ivoire (15)<br>Central African Republic (2)<br>Burkina Faso (1)<br>Cameroon (1)

2. WHATSAPP HOTLINE USAGE (over the reporting period)

**Total number of requests**	8 requests total
**Requests from treatment group households**	4 requests (2 questions about the program regarding bus tickets and 2 other questions about the monthly survey)
**Requests from control group households**	4 requests (2 questions about the program and 2 other questions)
**Requests from non-participant households**	0 requests
**# of bus tickets provided in the reporting period**	2 outbound tickets to Ghana<br>0 return tickets

3. CHANGES TO THE RISK ENVIRONMENT FOR PARTICIPANTS

**Campaign of anti-immigrant violence in destination countries**	From media tracking: nothing to report
**Severe outbreaks of COVID-19 in Niger or destination countries**	From media tracking: there have been no major outbreaks of COVID-19 in Niger or the destination countries.
**Severe political instability or political violence in destination countries**	From media tracking: extremist violence by jihadist militants continues in Mali, Burkina Faso, and Niger. Throughout the month of June, there have been numerous attacks by jihadists against both civilians and governmental armed forces in the Tillaberi and Diffa regions in Niger.<br><br>On June 9th, in Burkina Faso, armed men targeted and killed over 100 civilians in the Seytenga region, a northern town in the country close to the Niger border. This followed a similar attack the previous week in the same region by armed men killing 11 military police.

4. SEVERE ADVERSE EVENTS

**Instances of death of the participant, grievous bodily harm**	Nothing to report
**Instances of death of the subject's spouse or child**	Nothing to report
**Instances of wife/family loses home or land**	Nothing to report
**Instances of participant experiencing severe human rights abuses in destination**	Nothing to report

5. PLANNED PROGRAM ADAPTATIONS

**Survey outreach methods**	To increase our response rate in the next round of data collection, we are working with the survey firm to ensure the survey is implemented at times when people are most likely to be at home and test a wider variation in the time of calls. We will start Round 4 immediately after the Tabaski celebration, when most people are in their villages. Enumerators will also implement a protocol to systematically vary the time of day that people receive phone calls, varying morning, afternoon, and evening attempts. If most people are working in their fields during the day, we want to try to contact them in the evening when they are most likely to be home and available to speak on the phone. Round 4

	will be the first to systematically include evening calls to see if that increases response rates.

6. EAC RECOMMENDATIONS

**Overall Recommendation**	No Change / Adaptation Needed / Complete Stop
**Description**	Description & rationale
**Additional data/info requests from the PPM team**	

Next meeting date:

Appendix

Monthly Surveys

Round 3 of monthly surveys was conducted from June 11-26, 2022. This survey was conducted by phone to primary participants only. Enumerators attempted to reach 208 primary respondents. Two respondents were excluded because they had refused to participate in the previous survey round.

Response rates by treatment group are summarized in Table 1. We include two response rates: 1) response rates only among primary participants and 2) response rates for households including primary participants plus secondary contacts. We reached primary participants in 44% of households in the treatment group and for 55% of households in the control group. In an additional 15% of control households and 18% of treated households, we reached secondary contacts who gave us information about primary participants we were otherwise unable to contact. These additional responses enabled us to learn about the status of these participants even though we were unable to directly interview them.

Table 1: Response rates by treatment group for primary participants

**Treatment group**	**1. Response rate** ***Primary participant only***	**2. Response rate** ***Including secondary contacts***
Treatment (n=110)	44%	62%
Control (n=100)	55%	70%

Tables 2 and 3 report the locations of primary participants. Table 2 summarizes firsthand reports from primary participants.

Table 3 summarizes reports from their secondary contacts. Secondary contact reports were collected when we were unable to reach the primary participant. We only include secondary contact reports for participants we were not otherwise able to directly reach.

Table 2: Location reports by primary participants

country	treatment	control
Burkina Faso	1	0
Cote d'Ivoire	8	8
Libya	0	1
Niger	39	44
Other	0	1

Table 3: Location reports by secondary contacts

country	treatment	control
Cameroon	1	0
Cote d'Ivoire	7	1
Libya	0	2
Niger	6	7
Other	2	0

Table 4 summarizes responses to key questions in the monthly survey, with respondents divided into treatment and control groups. For each question, we report either the percentage of respondents in each group who gave a specified response or the mean response within the group. All respondents answered all questions reported in the table. Note that there are no statistically significant differences between treatment groups for any outcome in Table 4, based on joint F-tests.

Table 5 disaggregates these responses into movers (those who are in a different location than the last time they were surveyed) and non-movers (those in the same location) for the treatment group and the control group.

Table 4: Responses to key survey questions (Primary Respondents)

Question	Treatment Group N=48	Control Group N=54
Are you currently living in the location where you were last surveyed? (Percent responding "Yes")	79%	82%
**Current country of residence:** Niger	81%	82%
Côte d'Ivoire	17%	15%
Burkina Faso	2%	0%
Libya	0%	2%
Do you plan to travel to find work in the next month? (Percent responding "Yes")	31%	32%
Have you engaged in any work for payment in the last month? (Percent responding "Yes")	75%	82%
Average monthly income	32,013 CFA	29,318 CFA
In the past 7 days, have you cut the size of meals or skipped meals? (Percent responding "Yes")	19%	24%
In the past month, have you been physically attacked? (Percent responding "Yes") *This refers to any kind of physical aggression.*	15%	15%
In the past month, how often if ever have you felt unsafe walking in your neighborhood? (Percent responding "Yes")	0%	2%
**In the past month, have you experienced any of the following situations or been threatened with them in a workplace:** (Percent responding "Yes")		
Had passport or other papers taken away so that I could not leave an employer	0%	0%
Been prevented from contacting friends or family	0%	0%
Had my pay withheld by my employer	17%	13%
Been forced to pay off a debt to my employer before I could leave	0%	0%
Been locked up at night or otherwise physically restrained by my employer	0%	0%
Been forced to engage in illegal activities by employer	0%	0%

Table 5: Responses disaggregated for movers and non-movers

	Treatment Group N=48		Control Group N=54	
**Question**	**Moved since last survey N=10**	**Did not move N=38**	**Moved since last survey N=10**	**Did not move N=44**
**Current country of residence:** Niger	10%	100%	0%	100%
Cote d'Ivoire	80%	0%	80%	0%
Burkina Faso	10%	0%	0%	0%
Libya	0%	0%	10%	0%
Other	0%	0%	10%	0%
Do you plan to travel to find work in the next month? (Percent responding "Yes")	0%	40%	0%	39%
Have you engaged in any work for payment in the last month? (Percent responding "Yes")	90%	71%	100%	77%
Average monthly income	63,556 CFA	32,012 CFA	43,300 CFA	29,318 CFA
In the past 7 days, have you cut the size of meals or skipped meals? (Percent responding "Yes")	0%	24%	0	30%
In the past month, have you been physically attacked? (Percent responding "Yes")	20%	13%	20%	14%
In the past month, how often if ever have you felt unsafe walking in your neighborhood? (Percent responding "Yes")	0%	0%	10%	0%

In the past month, have you experienced any of the following situations or been threatened with them in a workplace: (Percent responding "Yes")				
Had passport or other papers taken away so that I could not leave an employer	0%	0%	0%	0%
Been prevented from contacting friends or family	0%	0%	0%	0%
Had my pay withheld by my employer	0%	21%	0%	16%
Been forced to pay off a debt to my employer before I could leave	0%	0%	0%	0%
Been locked up at night or otherwise physically restrained by my employer	0%	0%	0%	0%
Been forced to engage in illegal activities by employer	0%	0%	0%	0%

Discussion

One concerning development from Round 3 was an appreciable decline in the response rate among primary participants. The response rate among all primary participants was 60% in round 1, which increased to 70% in round 2, only to fall to 50% in round 3. However, despite our decreased overall response rate, we were able to increase the number of surveys with primary participants living abroad in both the treatment and control groups from round 2 to round 3. The response rate among these respondents increased by a factor of 5 from round 2 to round 3.[1]

By contrast, we were able to contact fewer primary participants who reported living in Niger in round 2.[2] This decrease may be due to our return to a phone-only data collection strategy, instead of our in person approach in round 2: 31 of the people we talked to in round 2 but not in round 3 had been interviewed in person. Another potential explanation for the decreased response rate in round 3 is the time of year. The survey was conducted during the labor-intensive planting season, when many people travel to work in fields a few kilometers away from their villages. They may leave their phones at home or be unable to charge them.

Respondents in Round 3 also reported being physically attacked at high rates: 15% of both treatment and control participants reported being physically attacked. Upon further investigation, there is no statistically significant difference between treatment groups, nor between people who traveled in the past month and those who did not. Nor do we observe differences among countries of residence. The individuals who reported physical attacks did not report feeling unsafe or forced labor situations. They were no more likely to report discrimination than other respondents. However, they were more likely to report being in poorer health compared to respondents who did not report suffering physical attacks. It is unfortunate and striking that respondents experienced physical assault at such high rates. This violence does not appear to be correlated with program participation or migration.

[1] In round 2, we only reached 4 primary participants who were abroad, but in round 3, we reached 20.
[2] 83 participants reported they were in Niger in round 3 compared to 97 in round 2.

F. Example: Recommendation from EAC.

EAC Recommendation to the PPM Team

Re: July 7, 2022 Monthly Report

Overall Recommendation: No Change

Description

The EAC reached a consensus agreement that the risk environment for participants has not significantly changed and therefore no change is currently required to the PPM pilot program.

The EAC members noted that the Round 3 survey response rates decreased, and look forward to learning more about the outcomes from additional tests to address the attrition concern after the next survey. EAC members recommended that the research team consider coordinating surveys with market days in each town/village as there is a higher change participants have their phone and a connection on those days.

EAC members note a concern about high rates of physical attack and would be interested in additional details on the nature of these attacks from future surveys.

EAC members encourage the PPM team to track security concerns in northern Benin, which is the area where Nigerien migrants may be interested in.

References

1. International Labor Organization, La mesure du travail décent au Niger (https://www.ilo.org/africa/information-resources/publications/WCMS_237000/lang--fr/index.htm) (2014) Accessed: 2021-12-05.
2. B Gado, J von der Goltz, M Saidi, AS Soumaila, Support to Jobs for Youth in Niger : A Retrospective Evaluation of Jobs Projects 2007-2018, (World Bank, Washington, DC), Technical Report Jobs Working Paper No. 34 (2019).
3. DAMOCLES Study Group, A proposed charter for clinical trial data monitoring committees: helping them to do their job well. *The Lancet* **365**, 711–722 (2005).
4. L Eckstein, Building a more connected dsmb: better integrating ethics review and safety monitoring. *Accountability research* **22**, 81–105 (2015).
